# C-C motif chemokine receptor 1 (CCR1) is a target of the EGF-AKT-mTOR-STAT3 signaling axis in breast cancer cells

**DOI:** 10.18632/oncotarget.21813

**Published:** 2017-10-10

**Authors:** Soon Young Shin, Da Hyun Lee, Jishin Lee, Chan Choi, Ji-Young Kim, Jeong-Seok Nam, Yoongho Lim, Young Han Lee

**Affiliations:** ^1^ Department of Biological Sciences, Sanghuh College of Life Sciences, Konkuk University, Seoul, Republic of Korea; ^2^ Cancer and Metabolism Institute, Konkuk University, Seoul, Republic of Korea; ^3^ Department of Pathology, Chonnam National University Medical School, Gwangju, Republic of Korea; ^4^ Laboratory Animal Resource Center, Gwangju Institute of Science and Technology, Gwangju, Republic of Korea; ^5^ School of Life Sciences, Gwangju Institute of Science and Technology, Gwangju, Republic of Korea; ^6^ Division of Bioscience and Biotechnology, BMIC, Konkuk University, Seoul, Republic of Korea

**Keywords:** CCR1, STAT3, AKT, mTOR, metastasis

## Abstract

The CC motif chemokine receptor 1 (CCR1) has been implicated in tumor invasion and metastasis in numerous cancers. However, the detailed mechanism of CCR1 upregulation in metastatic tumor cells is poorly understood. The aim of this study was to clarify the regulatory mechanism underlying transcriptional activation of the *CCR1* gene in response to epidermal growth factor (EGF) stimulation in breast cancer cells. CCR1 was highly expressed in human breast invasive ductal carcinoma (IDC) compared to adjacent normal tissues. Upon EGF stimulation, CCR1 expression was upregulated at the transcriptional level. Promoter analysis showed that signal transducer and activator of transcription 3 (STAT3) is necessary for EGF-induced *CCR1* promoter activation, and STAT3 silencing abrogated EGF-induced CCR1 transcription. Pharmacological inhibition and short hairpin RNA-mediated knockdown experiments showed that AKT-dependent mammalian target of rapamycin (mTOR) activation was involved in the phosphorylation of serine-727 of STAT3, which in turn stimulated the transcription of the *CCR1* gene. In conclusion, the AKT-mTOR-STAT3 signaling axis contributes to EGF-induced CCR1 expression, which promotes invasion and metastasis in breast cancer cells. We propose that the AKT-mTOR-STAT3 axis is a potential therapeutic target for blocking the invasion and metastasis of breast cancers.

## INTRODUCTION

Metastasis is the spread of cancer cells from the original tumor to other sites of the body through the tumor vasculature and lymphatics; for most solid tumors, it is the major cause of death. Despite our increasing knowledge on tumorigenesis and metastasis, our current understanding cannot fully explain the molecular mechanisms underlying the progression of metastasis.

The tumor microenvironment comprises infiltrating immune cells, endothelial cells, and stromal fibroblasts, which may provide the growth factors and cytokines that promote tumor growth, survival, and invasion [1]. It has been shown that cross-talk between tumor cells and stromal cells is required for the promotion and progression of cancer [2]. In the tumor microenvironment, stromal cells and cancer cells produce various chemokines that control tumor growth and progression [2–4], and emerging evidence shows that a variety of chemokine receptors are highly expressed on tumor cells and involved in tumor growth and metastasis [1, 4, 5]. In human breast cancer cells, chemokine receptors such as CXC chemokine receptor 4 (CXCR4) and CC motif chemokine receptor 7 (CCR7) are overexpressed and are involved in chemotactic and invasive responses [1, 5].

CCR1, a member of the seven-transmembrane G-protein-coupled receptor family, is widely expressed in many cell types and is involved in the activation and trafficking of immune cells, including neutrophils, monocytes, and lymphocytes [6]. CCR1 binds to multiple cytokines, including CCL3 (or MIP-1α), CCL5 (or RANTES), CCL7 (or MCP-3), CCL8 (or MCP-2), CCL14, CCL15 (or Leukotactin-1), CCL16, and CCL23 (or MPIF-1). CCR1 is highly expressed in diverse cancers, including prostate cancer cells, ovarian cancer, multiple myeloma, and hematolymphoid neoplasia [7–11]. Previous studies have demonstrated that CCR1 promotes the invasion of PC3 prostate cancer [7], hepatocellular carcinoma [12], ovarian cancer stem-like [9], and non-small cell lung cancer [13, 14] cells. These results indicate that CCR1 is associated with the progression of tumor invasion and metastasis [15]. In addition, CCR1 and its ligand CCL5 are highly expressed in ovarian cancer stem-like cells, suggesting the idea of a possible autocrine loop that maintains metastatic capability [9]. However, it remains largely unknown whether CCR1 is overexpressed in breast cancer tissues, and whether CCR1 promotes breast metastasis.

Epidermal growth factor (EGF) is a crucial factor in mammary gland development [16] and EGFR-targeted therapy provides some promising effects in patients with triple-negative (estrogen receptor-, progesterone receptor-, and erb-b2 receptor tyrosine kinase 2-negative) breast cancer [17]. In this study, we aimed to investigate the expression status of CCR1 in breast cancer tissues, the functional relevance of CCR1 in breast cancer metastasis, and the mechanisms underlying the regulation of CCR1 expression in breast cancer cells.

## RESULTS

### Higher expression of CCR1 in human breast cancer tissue

**Figure 1:**
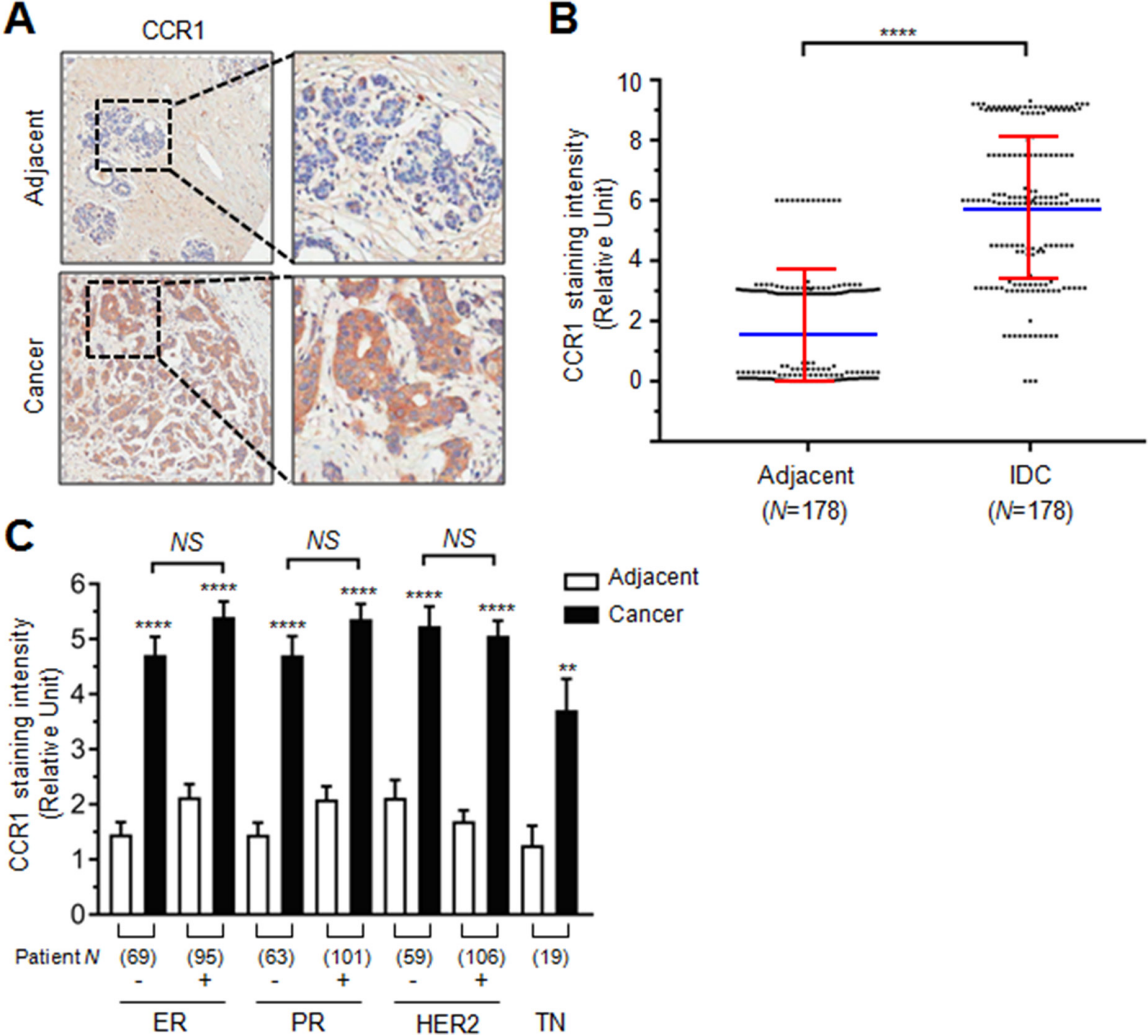
Role of CCR1 in the invasion and metastasis of breast cancer cells **(A)** Representative immunohistochemical staining of CCR1 in breast invasive ductal carcinoma (IDC) and adjacent normal tissue samples. Square *N* indicates total tissue number. **(B)** Relative staining scores of CCR1 immunoreactivity in IDC and adjacent normal tissues. Data are mean ± SD. Spot, individual sample; blue horizontal line, mean; red vertical line, error bar. ^****^, *P* < 0.0001 (n = 178, paired two-tailed *t*-test). **(C)** Relative immunohistochemical staining intensity of CCR1 in ER, PR, HER2, or TN negative (-) or positive (+) samples. Square number indicates total sample number. ER, estrogen receptor; PR, progesterone receptor; HER2, human epidermal growth factor receptor 2; TN, triple negative (ER^-^/PR^-^/HER2^-^). Values are expressed as mean ± SD. *NS*, not significant; ^****^, *P* < 0.0001; ^**^, *P* < 0.0220 by one-way ANOVA followed by Sidak’s multiple comparisons test (comparison between normal and cancer groups).

To investigate the role of CCR1 in human breast cancer, we first evaluated its expression in a tissue microarray composed of human breast invasive ductal carcinoma (IDC) and their adjacent peripheral normal tissues. We found that the CCR1 immunoreactivity was stronger in IDC than in adjacent normal tissues (Figure 1A). Relative CCR1 staining intensity was significantly higher in IDC than their adjacent normal tissues (mean ± SD; normal: 1.669 ± 1.886, Cancer: 5.781 ± 2.513; *P* < 0.0001 by two-tailed paired *t*-test, *n* = 178) (Figure 1B, Supplementary Table 1). Tissue microarray samples were composed of estrogen receptor (ER) negative and positive, progesterone receptor (PR) negative and positive, human epidermal growth factor receptor 2 (HER2) negative and positive, and triple negative (TN; ER^-^/PR^-^/HER2^-^) specimens. Statistical analysis shows that ER, PR, and HER2 expression was not associated with CCR1 staining intensity (Figure 1C). These data suggest that CCR1 is overexpressed in breast invasive ductal carcinoma (IDC) as compared to adjacent normal tissues, regardless of ER, PR, and HER2 expression status.

### Silencing of CCR1 attenuates the invasive and metastatic capabilities of MDA-MB-231 cells

**Figure 2:**
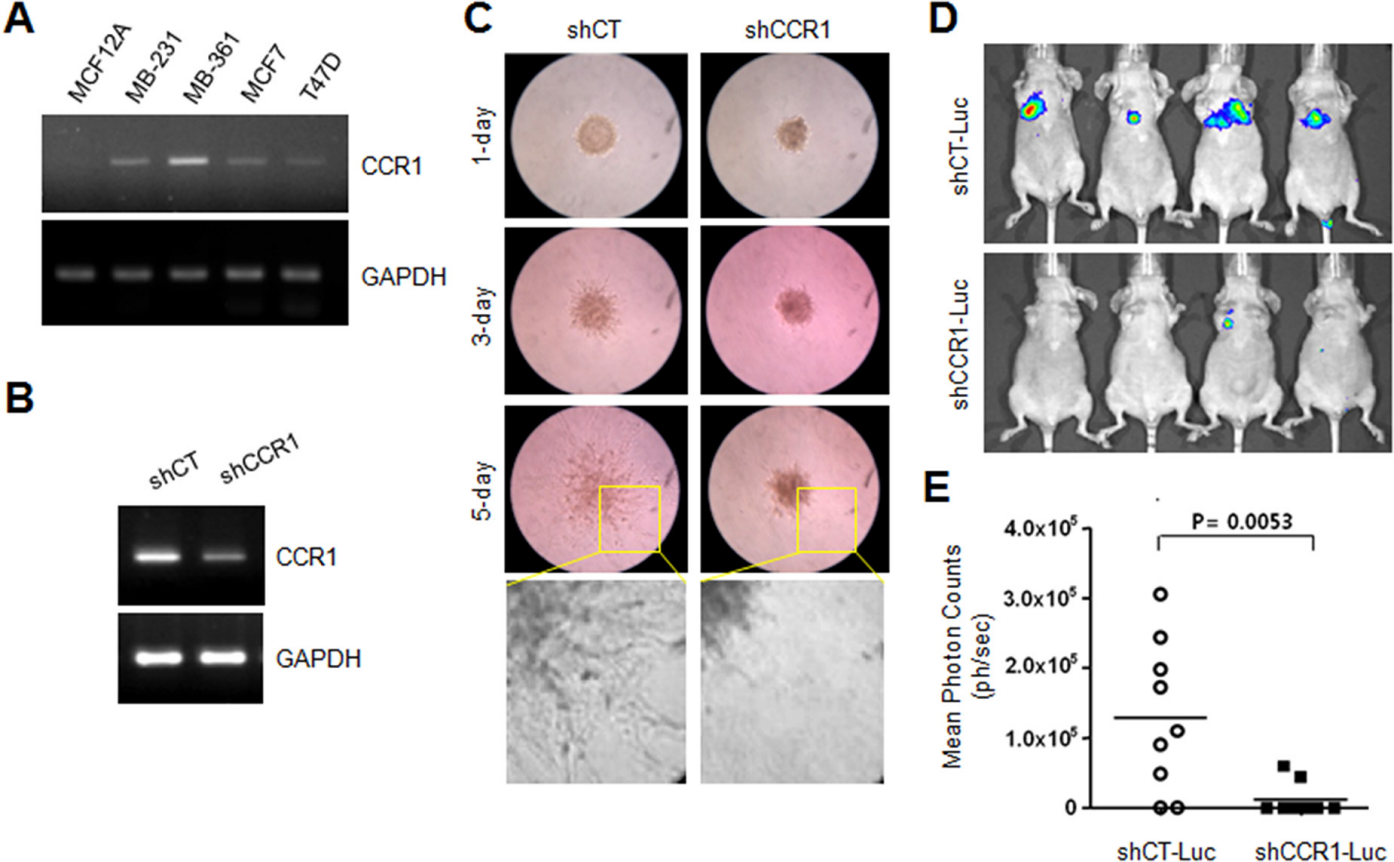
Effect of CCR1 silencing on experimental tumor invasion and metastasis **(A)** CCR1 expression in various breast cancer cells. Steady-state CCR1 mRNA levels were examined by RT-PCR. GAPDH was used as an internal control. **(B)** Silencing of CCR1 by expression of lentiviral CCR1 shRNA (shCCR1). Knockdown of CCR1 expression was verified by RT-PCR. **(C)** Invasion assay. MDA-MB-231 transfectants expressing shCT or shCCR1 were cultured in three-dimensional spheroids in an extracellular matrix. Protrusion of invasive cells was captured with an Eclipse TS100 microscope equipped with a digital sight camera. **(D)** Experimental *in vivo* lung metastasis. Representative BLI imaging of nude mice bearing MDA-MB-231 (shCT-Luc and shCCR1-Luc) tumors with metastatic lung lesions. **(E)** Eight weeks after inoculation, BLI signal intensity was measured in MDA-MB-231 transfectants with Spectrum IVIS.

Previous studies have demonstrated that CCR1 is involved in tumor metastasis in several cancers [9, 12–14]. However, the functional role of CCR1 is unclear in breast cancer metastasis. The steady-state levels of CCR1 mRNA were examined in various breast cancer cell lines. RT-PCR analysis showed that CCR1 mRNA levels were readily detectable in most tested breast cancer cell lines, but not in untransformed MCF12A breast epithelial cells (Figure 2A). For further characterization, we selected the MDA-MB-231 breast cancer cell line because these cells are highly metastatic and induce cell migration upon EGF stimulation [18].

To evaluate the functional role of CCR1 in the regulation of invasion, we analyzed the invasiveness of MDA-MB-231 cells expressing either a scrambled shRNA (shCT) or CCR1 shRNA (shCCR1). Knockdown of CCR1 was verified by RT-PCR (Figure 2B). Three-dimensional (3-D) spheroid culture demonstrated that control shCT cells showed high invasiveness out of the spheroid and into the surrounding matrix as spindle-like protrusions (Figure 2C). In contrast, CCR1-silenced cells showed an obvious reduction in invasive sprouting. To determine the effect of CCR1 silencing on metastatic properties *in vivo*, we injected luciferase-expressing MDA-MB-231 transfectants (shCT-Luc and shCCR1-Luc cells) into the tail veins of immunodeficient mice, and metastatic activity was determined by bioluminescence imaging (Figure 2D). At 8-weeks post-injection, mice injected with shCT-Luc cells had large tumors and increased bioluminescence signals. In contrast, shCCR1-Luc cells showed decreased lung metastatic ability in the mice. Whole-body imaging revealed that silencing of CCR1 significantly reduced the bioluminescence signal (Figure 2E). These data demonstrate that CCR1 promotes the invasion and metastasis of MDA-MB-231 breast cancer cells.

### EGF induces CCR1 expression in human breast cancer cells

**Figure 3:**
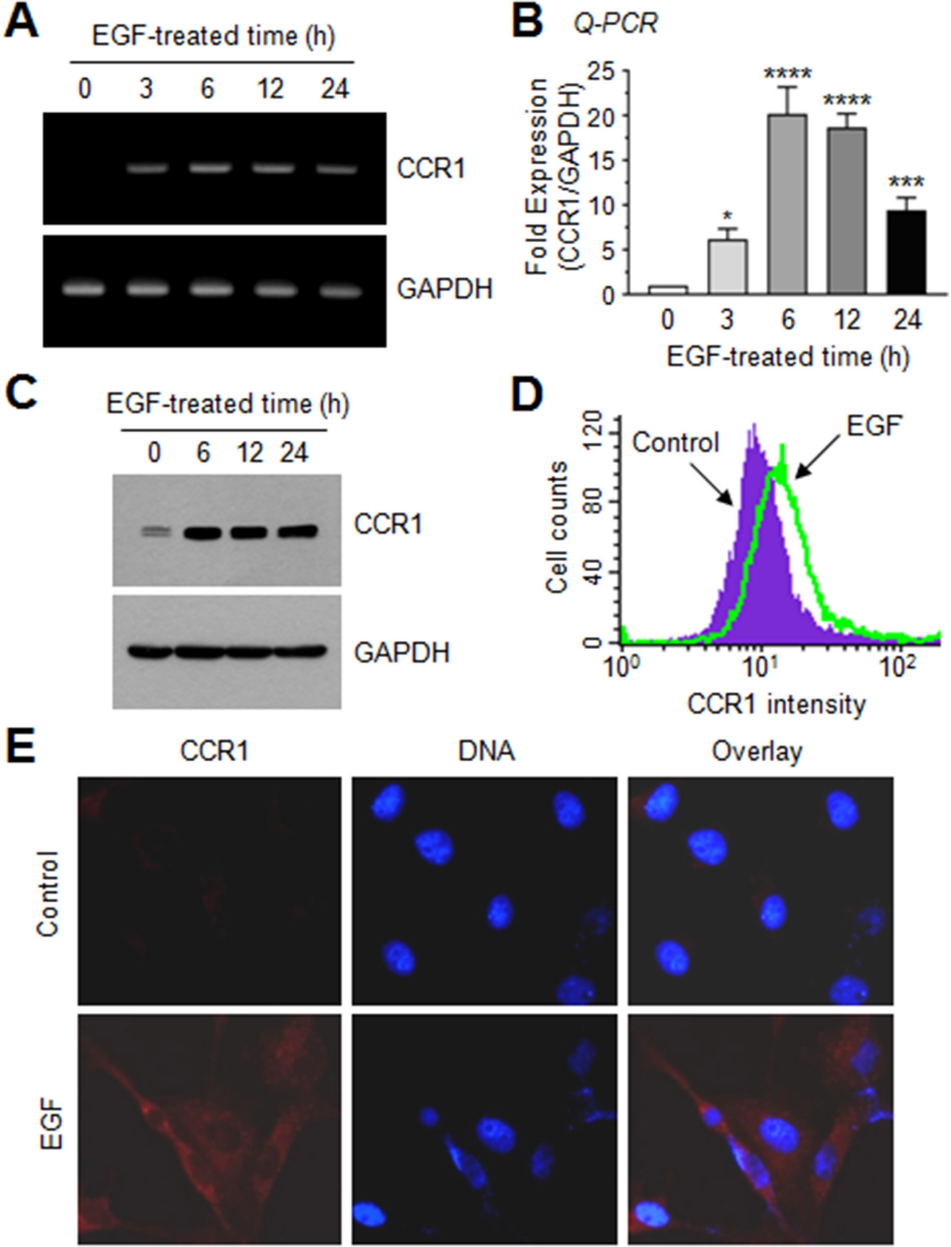
EGF enhances CCR1 expression in MDA-MB-231 breast cancer cells **(A)** MDA-MB-231 cells were serum-depleted overnight and treated with EGF (50 ng/mL) for 0–24 h, and total RNA was extracted. CCR1 mRNA levels were examined by RT-PCR. GAPDH was used as an internal control. **(B)** Serum-starved MDA-MB-231 cells were treated as in A, and CCR1 mRNA levels were assessed by quantitative real-time PCR (*Q-PCR*). Values were normalized to GAPDH mRNA levels. Data are mean ± SD (*n =* 3). ^*^, *P =* 0.0247; ^***^, *P* < 0.0009; ^****^, *P* < 0.0001 (compared to untreated control; by Sidak’s multiple comparisons test). **(C)** Serum-starved MDA-MB-231 cells were treated as in A, and CCR1 protein levels were assessed by immunoblotting. GAPDH was used as an internal control. **(D)** Flow cytometry. After serum-starved MDA-MB-231 cells were treated with EGF (50 ng/mL) for 12 h, CCR1 protein expression on the cell surface was measured using flow cytometry. **(E)** MDA-MB-231 cells were treated with EGF (50 ng/mL) for 12 h, and then incubated with an antibody against CCR1, and a AlexaFluor 555-conjugated (*red signal*) secondary antibody for 30 min. Nuclear DNA was stained with 0.1 μg/mL Hoechst 33258 for 10 min (*blue signal*). Fluorescence-positive cells were examined under an EVOSf1^®^ fluorescence microscope.

To investigate the molecular mechanism underlying *CCR1* gene regulation, we tested whether EGF up-regulates CCR1 expression in MDA-MB-231 cells by RT-PCR. After serum starvation, CCR1 mRNA was undetectable; however, after EGF stimulation for 3 h, CCR1 mRNA expression was increased (Figure 3A). Quantitative real-time PCR (Q-PCR) demonstrated maximum CCR1 mRNA expression after approximately 6 h, with 20.2- and 18.7-fold increases at 6 and 12 h, respectively, compared to the basal level (Figure 3B). Elevated CCR1 protein levels were confirmed by immunoblotting (Figure 3C), flow cytometry (Figure 3D), and immunofluorescence microscopy (Figure 3E). Thus, EGF facilitates CCR1 expression in highly metastatic MDA-MB-231 cells.

### Signal transducer and activator of transcription (STAT)-binding element stimulates EGF-induced CCR1 promoter activation

**Figure 4:**
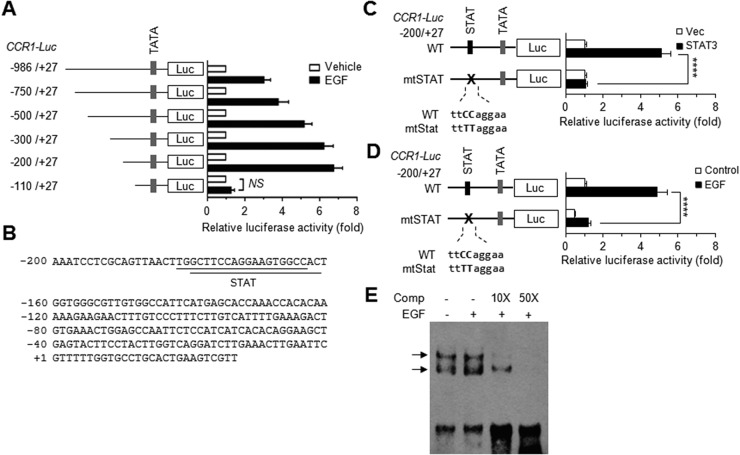
EGF induces CCR1 expression via STAT3 **(A)** MDA-MB-231 cells were transfected with 0.2 μg of the promoter-reporter plasmids along with 50 ng of the *Renilla* luciferase expression plasmid, pRL-null, for normalization of transfection efficiency. After 48 h, the cells were either treated with EGF (20 ng/mL) for 12 h or untreated (control), after which dual luciferase activity was measured. Data are means ± SD (*n =* 3). **(B)** The nucleotide sequence of the promoter region (−200 to +27) of human *CCR1*. **(C)** MDA-MB-231 cells were transfected with either wild-type pCCR1-Luc(−200/+27) or a STAT site mutant construct (mtSTAT), and with either an empty vector or a STAT3 expression plasmid. After 48 h, the cells were collected, and dual luciferase activity was measured. Data are means ± SD (*n =* 3). ^****^, *p* < 0.0001. **(D)** MDA-MB-231 cells were transfected with either wild-type pCCR1-Luc(−200/+27) or mtSTAT. After 48 h, the cells were either treated with EGF (20 ng/mL) for 12 h or untreated, and then, dual luciferase activity was measured. Data are means ± SD (*n =* 3). ^****^, *p* < 0.0001 by Sidak’s multiple comparisons test. **(E)** EMSA. MDA-MB-231 cells were treated with EGF (50 ng/mL) for 12 h or untreated, and nuclear extracts were prepared. EMSA was performed with biotin-labeled oligonucleotide probes containing the STAT3 binding sequence (5′-gttaacttggcttccaggaagtggc-3′) in the absence or presence of a 10-fold (10*×*) or 50-fold (50*×*) molar excess of unlabeled competitor oligonucleotides (*Comp*).

To determine the transcriptional mechanism regulating CCR1 expression, a series of 5′-deletion constructs of human *CCR1* were generated and transfected into MDA-MB-231 cells. The data revealed that these deletions affected EGF stimulation-induced *CCR1* promoter activity (Figure 4A). Notably, deletion of the –200 to –111 region dramatically decreased EGF-induced *CCR1* promoter activity, suggesting that this region is crucial for EGF-induced *CCR1* transcription.

Using the web-based transcription factor search tool MatInspector (http://www.genomatix.de/), we identified two overlapping binding sites of signal transducer and activator of transcription (STAT) at –183/–165 and –181/–162 bp in the *CCR1* promoter (Figure 4B). To determine the possible contribution of these STAT-binding elements to EGF-induced *CCR1* transcription, the core sequence of the STAT-binding motif at –183/–162 was mutated within the –200/+27 construct (mtStat; ttCCagga → ttTTagga) by site-directed mutagenesis (Figure 4C). Transient transfection of STAT3 *trans*-activated the wild-type construct but not the STAT mutant construct. Notably, disruption of the STAT sites caused a significant reduction in EGF responsiveness (*p* < 0.0001) compared to the wild-type construct (Figure 4D). We next determined whether the STAT-binding elements are responsive to EGF stimulation. An electrophoretic mobility shift assay (EMSA) showed that EGF increased the amount of DNA-protein complexes, which were reduced by the addition of excess unlabeled STAT-binding oligonucleotides (Figure 4E). These data suggest that STAT3 binds to the STAT binding motifs in the *CCR1* promoter region and is responsible for EGF signaling-mediated *CCR1* transcription.

### Inhibition of STAT3 reduces EGF-induced *CCR1* transcription

**Figure 5:**
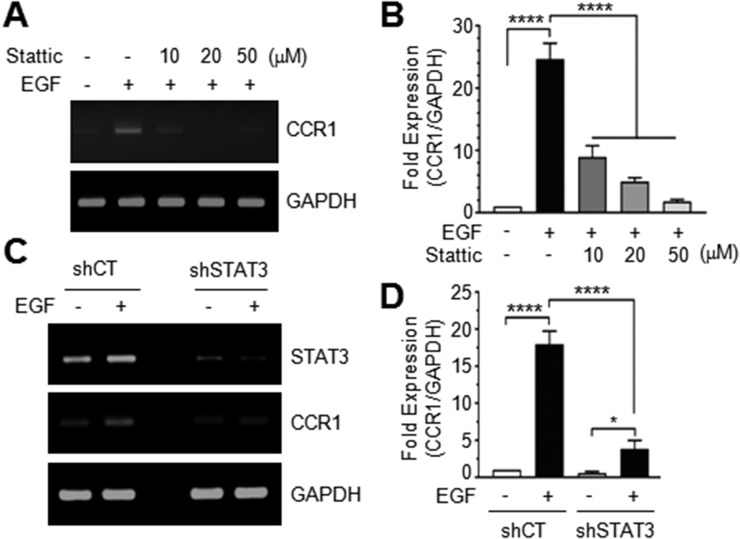
Effect of STAT3 silencing on CCR1 expression **(A)** Serum-starved MDA-MB-231 cells were treated with EGF (50 ng/mL) in the absence or presence of different concentrations of Stattic. After 12 h, total RNA was extracted, and CCR1 mRNA levels were examined by RT-PCR. **(B)** Serum-starved MDA-MB-231 cells were treated as in A, and CCR1 mRNA levels were assessed by quantitative real-time PCR. Values were normalized to GAPDH mRNA levels. Data are means ± SD (*n =* 3). ^****^, *p* < 0.0001 by Sidak’s multiple comparisons test. **(C)** Serum-starved MDA-MB-231 transfectants expressing control scrambled shRNA (shCT) or STAT3 shRNA (shSTAT3) were treated with EGF (50 ng/mL) for 12 h, and total RNA was extracted. CCR1 mRNA levels were examined by RT-PCR. GAPDH was used as an internal control. **(D)** Cells were treated as in C, and CCR1 mRNA levels were determined by quantitative real-time PCR. Data are means ± SD (*n =* 3). ^*^, *p =* 0.0157; ^****^, *p* < 0.0001 by Sidak’s multiple comparisons test.

Pretreatment of MDA-MB-231 cells with Stattic, a small molecule inhibitor of STAT3, dose-dependently decreased EGF-induced CCR1 mRNA accumulation (Figure 5A and 5B). To further address the role of STAT3, we established MDA-MB-231 transfectants stably expressing scrambled control shRNA (shCT) or STAT3 shRNA (shSTAT3). Reduction of STAT3 expression was confirmed by RT-PCR. Knockdown of endogenous STAT3 reduced EGF-induced CCR1 mRNA expression (Figure 5C). EGF stimulation increased CCR1 mRNA levels 22.1-fold in MDA-MB-231/shCT cells, but only 3.5-fold increase in MDA-MB-231/shSTAT3 cells (Figure 5D). Similarly, knockdown of STAT3 in T24 bladder cancer cells (Supplementary Figure 1A and 1B) and Capan-1 pancreatic cancer cells (Supplementary Figure 1C and 1D) dramatically decreased CCR1 mRNA levels, as revealed by RT-PCR and Q-PCR. These findings suggest that STAT3 mediates EGF-induced CCR1 expression and that the regulatory function of STAT3 in CCR1 expression is not limited to breast cancer cells.

### Inhibition of AKT suppresses EGF-induced serine-727 phosphorylation of STAT3 and CCR1 expression

**Figure 6:**
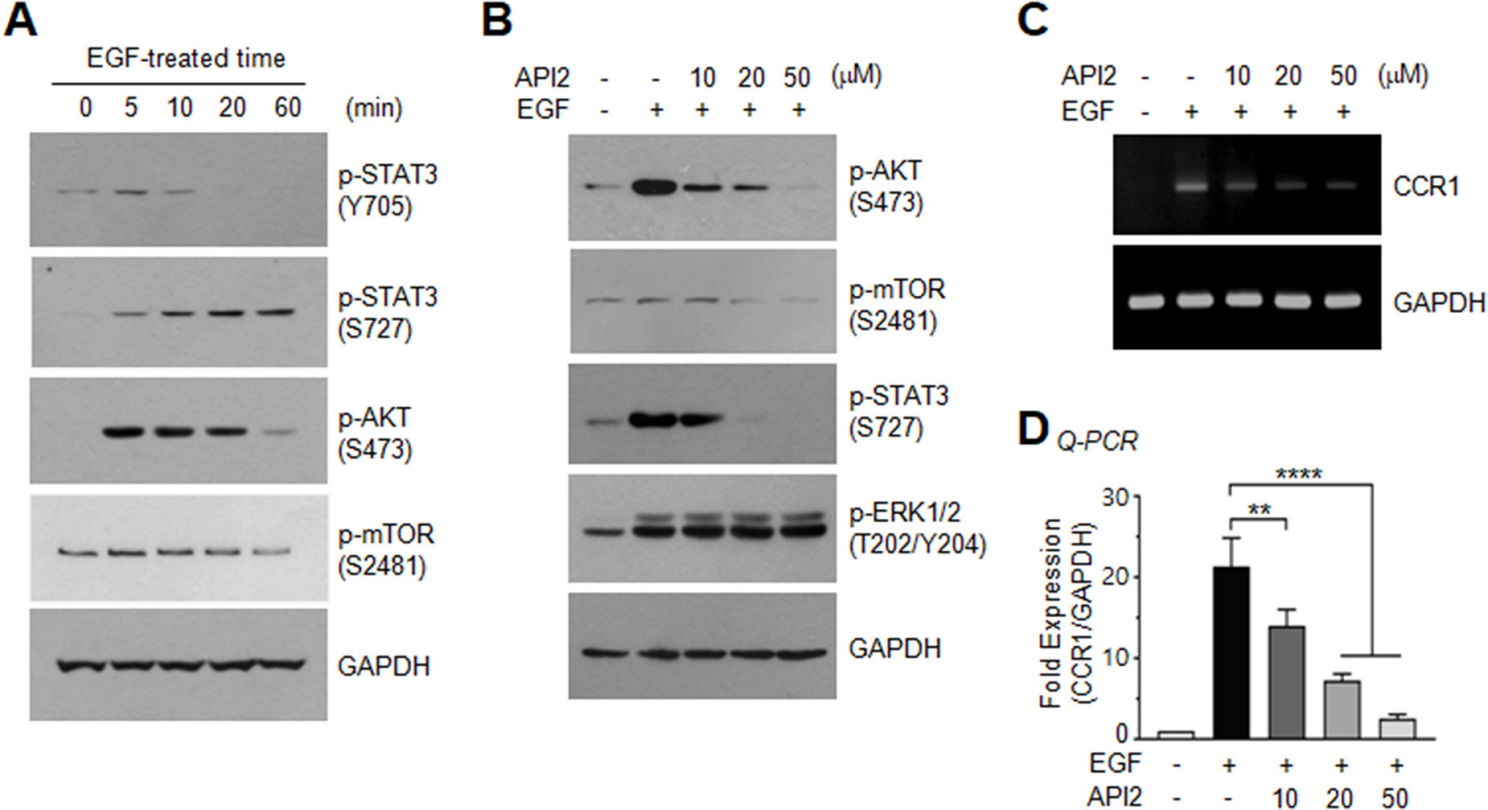
Effect of AKT inhibition on EGF-induced STAT3 phosphorylation and CCR1 expression **(A)** Serum-starved MDA-MB-231 cells were treated with EGF (50 ng/mL) for different times, and phosphorylation of STAT3 (Tyr705), STAT3 (Ser727), AKT (Ser473), or mTOR (Ser2481) was analyzed by immunoblotting. GAPDH was used as an internal control. **(B)** Serum-starved MDA-MB-231 cells were either pretreated with API2 (10, 20, or 50 μM) for 30 min or untreated, and then treated with EGF (50 ng/mL). After 15 min, the cells were collected, and protein lysates were prepared. Phosphorylation of AKT (Ser473), mTOR (Ser2481), STAT3 (Ser727), or ERK1/2 (Thr201/Tyr204) was analyzed by immunoblotting. GAPDH was used as an internal control. **(C)** Cells were treated as in (B). After 24 h, total RNAs were extracted, and CCR1 mRNA levels were examined by RT-PCR. GAPDH was used as an internal control. **(D)** Serum-starved MDA-MB-231 cells were treated as in B, and CCR1 mRNA levels were assessed by quantitative real-time PCR (*Q-PCR*). Values were normalized to GAPDH mRNA levels. Data are means ± SD (*n =* 3). ^**^, *p* = 0.0027; ^****^, *p* < 0.0001 by Sidak’s multiple comparisons test.

To investigate the STAT3-stimulating signaling pathway responsible for CCR1 expression, serum-starved MDA-MB-231 cells were treated with EGF (50 ng/mL). In accordance with a previous study [19], we observed an increase in STAT3 phosphorylation by EGF; of note, tyrosine-705 phosphorylation was transient, while serine-727 phosphorylation was long-lasting over 60 min (Figure 6A), suggesting that serine phosphorylation of STAT3 may play a role in CCR1 expression. We next sought to identify the serine kinase that phosphorylates serine-727 of STAT3. One possible candidate is AKT, which is important for tumor initiation [20] and metastasis [21]. AKT and its downstream effector, mammalian target of rapamycin (mTOR), were rapidly phosphorylated by EGF (Figure 6A). To determine whether AKT participates in EGF-induced CCR1 expression, we utilized the pharmacological AKT inhibitor, API2 [22]. We confirmed that API2 inhibited EGF-induced AKT phosphorylation (Figure 6B). API2 also inhibited EGF-induced mTOR phosphorylation, as well as phosphorylation of serine-727 of STAT3, but had no effect on ERK1/2 phosphorylation (Figure 6B). In addition, the EGF-induced accumulation of CCR1 mRNA was decreased by API2 in a dose-dependent manner as revealed by RT-PCR (Figure 6C) and Q-PCR (Figure 6D). These data suggest that AKT signaling is involved in EGF-induced CCR1 mRNA expression.

### Silencing of AKT inhibits EGF-induced serine-727 phosphorylation of STAT3 and CCR1 expression

**Figure 7:**
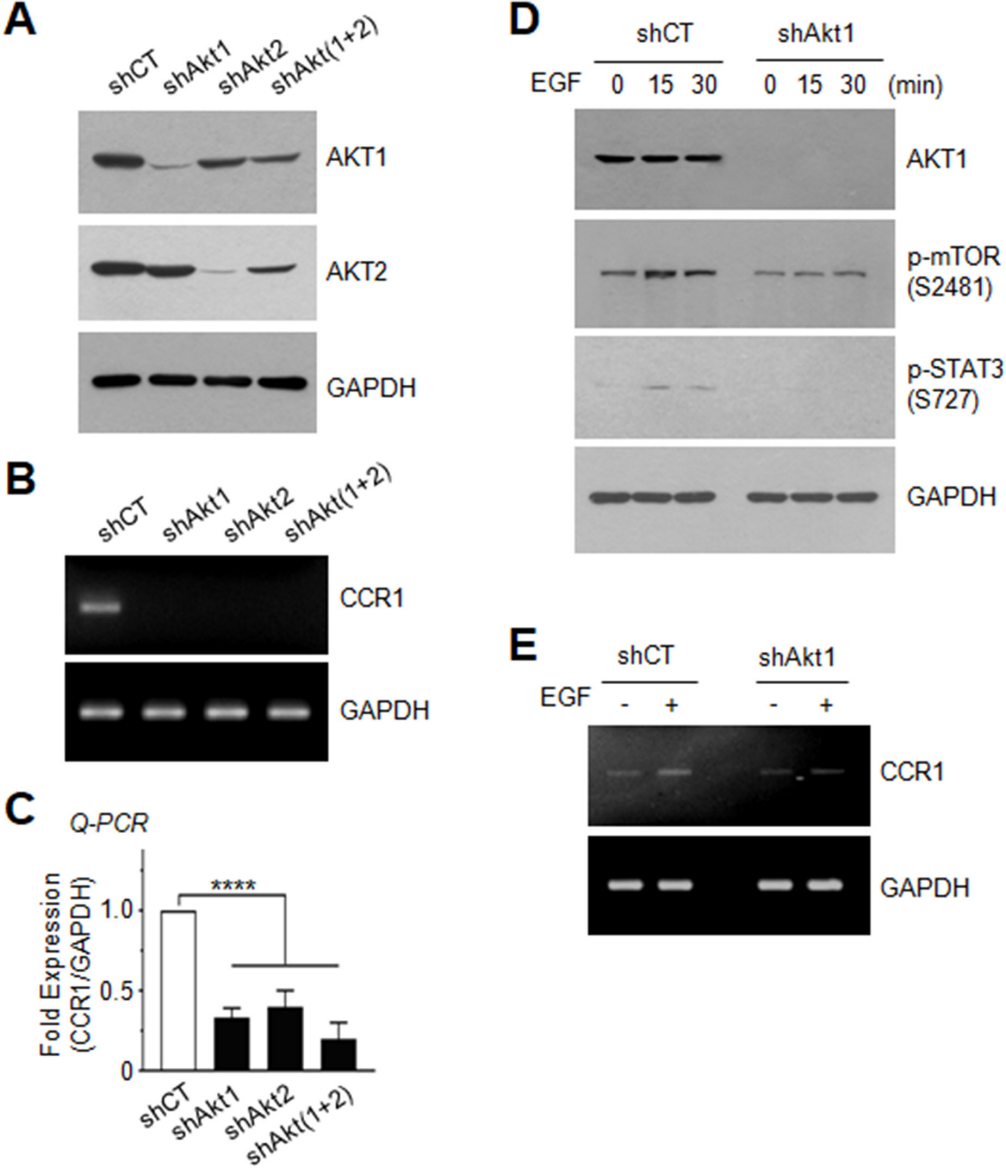
Effect of AKT silencing on EGF-induced STAT3 phosphorylation and CCR1 expression **(A)** AKT1 or AKT2 expression was examined in MDA-MB-231 cells expressing control shRNA (shCT), AKT1 shRNA (shAkt1), AKT2 shRNA (shAkt2), or AKT1 plus AKT2 [shAkt(1+2)] shRNAs. GAPDH was used as an internal control. **(B)** CCR1 mRNA expression was detected by RT-PCR in shCT, shAkt1, shAkt2, or shAkt(1+2)-expressing cells. GAPDH was used as an internal control. **(C)** CCR1 mRNA expression was detected by Q-PCR in shCT, shAkt1, shAkt2, or shAkt(1+2)-expressing cells. Values were normalized to GAPDH mRNA levels. Data are means ± SD (*n =* 3). ^****^, *p* < 0.0001 by Sidak’s multiple comparisons test. **(D)** Serum-starved shCT or shAkt1 cells were either untreated or treated with EGF (50 ng/mL) for 15 or 30 min, and the phosphorylation status of mTOR (Ser2481) and STAT3 (Ser727) was analyzed by immunoblotting. GAPDH were used as internal controls. **(E)** Serum-starved shCT or shAkt1 cells were treated with EGF (50 ng/mL) for 24 h, and CCR1 mRNA expression was detected by RT-PCR. GAPDH was used as an internal control.

To further address the possible involvement of AKT in the regulation of CCR1 expression, we established MDA-MB-231 transfectants in which AKT1, AKT2, or AKT1 plus AKT2 [AKT(1+2)] were stably knocked down using a lentiviral shRNA delivery system (Figure 7A). Silencing of AKT1, AKT2, or AKT(1+2) led to decreased expression of CCR1 mRNA, as revealed by RT-PCR (Figure 7B) and Q-PCR (Figure 7C). Furthermore, AKT1 silencing resulted in the inhibition of EGF-induced phosphorylation of mTOR at serine-2481 and STAT3 at serine-727 (Figure 7D), as well as CCR1 mRNA expression (Figure 7E). These data demonstrate that AKT stimulates STAT3, leading to up-regulation of CCR1 mRNA expression. Similar results were observed for STAT3 phosphorylation on serine-727 (Supplementary Figure 2A) and CCR1 mRNA expression (Supplementary Figure 2B and 2C) in T24 bladder and Capan-1 pancreatic cancer cells expressing AKT1 shRNA.

To extend our analysis of the role of AKT in CCR1 expression, we tested whether exogenous expression of AKT increases CCR1 expression. Exogenous expression of V5-tagged AKT1 in non-transformed MCF12A breast epithelial cells was confirmed by immunoblotting (Supplementary Figure 3A). We selected two transfectants (clones #1 and #2), and measured CCR1 mRNA expression. CCR1 mRNA levels were strongly increased by exogenous AKT1 expression compared with that in empty vector-transfected cells (Supplementary Figure 3B and 3C). These data demonstrated that AKT mediates EGF-induced CCR1 mRNA expression through activation of STAT3.

### mTOR is necessary for AKT-induced serine-727 phosphorylation of STAT3 and CCR1 expression

**Figure 8:**
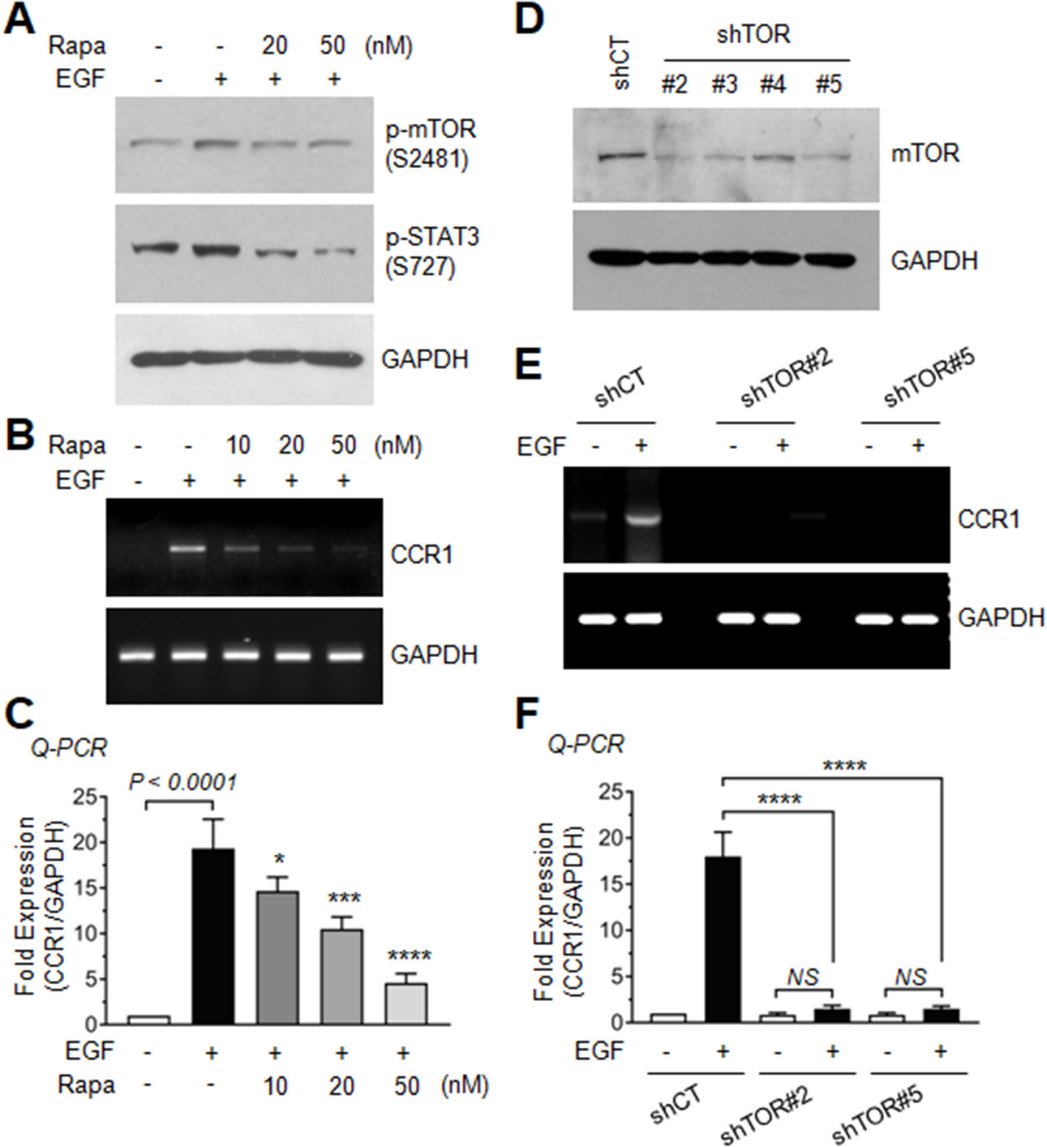
Role of AKT-dependent mTOR activation in EGF-induced CCR expression **(A)** Serum-starved MDA-MB-231 cells were either left untreated or pretreated with rapamycin (20 or 50 nM) for 30 min, and then treated with EGF (50 ng/mL). After 15 min, the cells were collected, and protein lysates were prepared. Lysates were immunoblotted with phospho-mTOR (Ser2481) and phospho-STAT3 (Ser727) antibodies. GAPDH was used as an internal control. **(B)** Serum-starved MDA-MB-231 cells were treated as in A. After 24 h, total RNAs were extracted, and CCR1 mRNA levels were examined by RT-PCR. GAPDH was used as an internal control. **(C)** Serum-starved MDA-MB-231 cells were treated as in B, and CCR1 mRNA levels were assessed by quantitative real-time PCR (*Q-PCR*). Values were normalized to GAPDH mRNA levels. Data are means ± SD (*n =* 3). ^*^, *p* = 0.0343; ^***^, *p* = 0.0004; ^****^, *p* < 0.0001 (compared with EGF-treated cells; by Sidak’s multiple comparisons test). **(D)** Silencing of mTOR by expression of lentiviral mTOR shRNA (shTOR). Knockdown of mTOR expression was verified by immunoblotting. **(E)** Serum-starved shCT and shTOR clones #2 and #5 cells were treated with EGF (50 ng/mL) for 24 h, and CCR1 mRNA expression was detected by RT-PCR. GAPDH was used as an internal control. **(F)** Serum-starved shCT and shTOR clones #2 and #5 cells were treated as in E, and CCR1 mRNA levels were assessed by Q-PCR. Values were normalized to GAPDH mRNA levels. Data are means ± SD (*n =* 3). ^****^, *p* < 0.0001; *NS*, statistically not significant (by Sidak’s multiple comparisons test).

A previous study has reported that inhibition of mTOR prevents the migration and invasion of leukemia cells through the inhibition of STAT3 phosphorylation [23]. We next asked whether EGF-induced mTOR activation is functionally linked to serine-727 phosphorylation of STAT3 and CCR1 expression. When MDA-MB-231 cells were treated with the mTOR inhibitor rapamycin, EGF-induced mTOR phosphorylation and STAT3 serine-727 phosphorylation (Figure 8A), as well as CCR1 mRNA expression (Figure 8B and 8C) were substantially decreased, suggesting that the rapamycin-sensitive mTOR/Raptor complex (mTORC1) is involved in EGF-induced phosphorylation of STAT3 and CCR1 mRNA expression.

To confirm the requirement of mTOR for EGF-induced CCR1 expression, we established MDA-MB-231 transfectants stably expressing mTOR shRNA (shTOR). Silencing of mTOR was confirmed by immunoblotting, and two clones (clones #2 and #5) were selected for further experiments (Figure 8D). Both two clones exhibited inhibition of EGF-induced CCR1 mRNA expression as revealed by RT-PCR (Figure 8E) and Q-PCR (Figure 8F). These data suggest that mTOR participates in serine-727 phosphorylation of STAT3 and CCR1 mRNA expression in response to EGF stimulation in MDA-MB-231 breast cancer cells.

## DISCUSSION

Invasion and metastasis are central to cancer development and progression. EGF stimulates the growth, proliferation, differentiation, and survival of diverse cell types and facilitates cellular migration. Amplification or uncontrolled activation of EGFR signaling has been implicated in the invasion and metastasis of many cancers. In breast cancer cells, inhibition of EGFR signaling reduces breast cancer cell invasion [24]. CCR1 is involved in the invasion and metastasis of several cancer types [14, 15]. Here, we show that EGF up-regulates CCR1 expression, suggesting that CCR1 may contribute to EGFR-mediated tumor invasion and metastasis. CCR1 expression is enhanced by many extracellular agents, including histamine [25], conditional medium collected from TNFα-activated mesenchymal stem cells [26], interleukin-2 [27], and insulin-like growth factor (IGF)-1 [28]. However, the mechanism underlying CCR1 upregulation in metastatic tumor cells is poorly characterized. Here, we found that EGF induces *CCR1* transcription by stimulating promoter activity, suggesting positive cross-talk between EGFR and CCR1 signaling. In this study, the transcriptional regulatory mechanism of the human *CCR1* gene was investigated using MDA-MB-231 breast cancer cells. The major findings of our study can be summarized as follows: (i) elevated expression of CCR1 in breast IDC; (ii) EGF-induced transcriptional upregulation of CCR1 expression; (iii) overlapping consensus STAT3-binding sequences (between –183 and –162) in the *CCR1* promoter are essential for EGF-induced *CCR1* transcriptional activation; (iv) knockdown of CCR1 or STAT3 expression reduces the invasive and metastatic capability of MDA-MB-231 cells; and (v) the PI3K-AKT-mTOR-STAT3 axis is required for EGF-induced CCR1 mRNA expression.

We observed that CCR1 mRNA was abundantly expressed in various breast cancer cell lines, including MDA-MB-231, MDA-MB-361, MCF7, and T47D cells, as compared with the levels in normal MCF12A breast epithelial cells. We further found that CCR1 mRNA expression in MDA-MB-231 cells was enhanced upon EGF stimulation, as revealed by RT-PCR, Q-PCR, fluorescence microscopy, and flow cytometry. In an effort to reveal the transcriptional regulatory mechanism of EGF-induced CCR1 expression, we identified the *cis*-acting response elements in the *CCR1* promoter. Using serial deletion constructs, we found that the region between –200 and –100 was required for EGF-induced *CCR1* promoter activity, and sequence analysis revealed two overlapping STAT3 elements between –183 and –162. The functional significance of these STAT3 binding sites was demonstrated by their mutation, which led to significantly reduced STAT3- and EGF-induced promoter activity. In addition, transfection of MDA-MB-231 cells with a STAT3-specific shRNA strongly attenuated EGF-induced *CCR1* transcription. STAT3 is ubiquitously expressed in most tissues, however, constitutively active STAT3 may contributes to tumor progression and metastasis in diverse cell types [29–32]. Our data clearly indicate that CCR1 silencing inhibited the invasive capability of MDA-MB-231 cells, as revealed in 3-D spheroid culture, and significantly inhibited lung metastasis, as revealed in an *in vivo* experiment. These results suggest that CCR1 promotes the metastasis of breast cancer cells. Thus, we hypothesized that STAT3 contributes to tumor invasion and metastasis by regulating CCR1 expression. These findings extend the function of EGFR in the promotion of invasiveness via up-regulation of CCR1 expression.

Another key finding of this study is that CCR1 expression is induced by the AKT-mTOR-STAT3 axis upon EGF stimulation. For maximal STAT3 activation, phosphorylation at both tyrosine and serine residues is required [33]. Tyrosine-705 phosphorylation of STAT3 is prerequisite for homodimerization, nuclear translocation, and DNA-binding activity [34], whereas serine-727 phosphorylation is necessary for maximal transcriptional activity [29, 35]. Tyrosine-705 is phosphorylated by several tyrosine kinases, such as EGFR, Src, and JAK family kinases [36, 37], and serine-727 phosphorylation by multiple serine kinases, including MAPKs (p38, ERK, JNK), PKCδ, mTOR, and NLK, independently of tyrosine-705 phosphorylation [35, 38, 39]. In MDA-MB-231 cells, we observed that EGF-induced tyrosine-705 phosphorylation rapidly declined after 10 min, whereas serine-727 phosphorylation was long lasting (up to 60 min). Here, we focused on the serine kinases involved in serine-727 phosphorylation of STAT3 in the regulation of CCR1 expression. Among the downstream effectors of EGFR, PI3K and AKT activities are significantly increased in triple-negative (ERα^-^/PR^-^/HER2^-^) breast cancers [40, 41], and PI3K expression is associated with poor prognosis in breast cancer [42]. In addition, targeting EGFR/PI3K/AKT pathway significantly inhibited migration and invasion of MDA-MB-231 cells [18]. Here we showed that AKT was highly phosphorylated within 5 min after EGF stimulation, and treatment with API2, a pharmacological inhibitor of AKT, reduced EGF-induced STAT3 phosphorylation and CCR1 mRNA expression. Moreover, shRNA-mediated silencing of AKT1 or AKT2 in MDA-MB-231 cells strongly attenuated STAT3 phosphorylation at serine-727 and CCR1 mRNA expression. Silencing of AKT1 in T24 bladder carcinoma cells and Capan-1 pancreatic carcinoma cells yielded similar results, suggesting that CCR1 expression activation via the AKT-STAT3 pathway is not restricted to breast cancer cells. However, in diethyl nitrosamine-induced mouse hepatocellular carcinoma cells, treatment with the PI3K inhibitor LY294002 had no effect on STAT3 phosphorylation at serine-727 [43]. Thus, it is unclear whether the AKT-STAT3 axis drives transcriptional activation of *CCR1* in diverse cancer cell types.

In summary, we propose a signaling mechanism by which EGF up-regulates the expression of CCR1 through the AKT-mTOR-STAT3 axis in MDA-MB-231 breast cancer cells. It will be of great interest to determine whether targeting of AKT-mTOR-STAT3 axis-dependent CCR1 expression can be a potential therapeutic strategy for the management of triple-negative breast cancer.

## MATERIALS AND METHODS

### Cells

Human breast non-transformed epithelial (MCF12A) and cancer cells (MDA-MB-231), bladder carcinoma (T24) and pancreatic adenocarcinoma (Capan-1) cells were obtained from the American Type Culture Collection (Manassas, VA, USA).

### Human breast cancer specimens and immunohistochemical analysis

A human breast tissue microarray, containing 189 breast cancer specimens composed of invasive ductal carcinoma (189 cases) and matched peripheral normal tissues, was generated by the Chonnam National University Hwasun Hospital National Biobank of Korea, a member of the National Biobank of Korea. Samples of invasive ductal carcinoma (IDC) and adjacent normal tissues (178 cases) were analyzed for CCR1 expression. Clinicopathologic parameters, including age at diagnosis, histopathologic type, tumor size, and nodal status, were evaluated. All IDC patients had not received chemotherapy or radiotherapy. All specimens were obtained after obtaining informed consent under an institutional review board (IRB)-approved protocol of the Chonnam National University Hwasun Hospital, Korea.

Immunohistochemical staining was performed with a Bond-Max autostainer system (Leica Microsystems, Bannockburn, IL, USA) using primary antibodies against CCR1 (1:150; Novus Biologicals, Littleton, CO, USA). All steps, which are listed below, were performed by the autostainer instrument, according to the manufacturer’s instructions, and all solutions were from Leica Microsystems: (i) deparaffinization of the tissue slides with Bond Dewax Solution at 72°C for 30 min, (ii) heat-induced epitope retrieval (antigen unmasking) with Bond Epitope Retrieval Solution 1 for 20 min at 100°C, (iii) peroxide blocking of the slides for 5 min at ambient temperature, (iv) incubation with the CCR1 primary antibody for 15 min at ambient temperature, (v) incubation with Post Primary reagent for 8 min at ambient temperature, followed by washing with Bond Wash solution for 6 min, (vi) incubation with Bond Polymer for 8 min at ambient temperature, followed by washing with Bond Wash and distilled water for 4 min, (vii) color development with 3,3′-diaminobenzidine tetrahydrochloride (DAB) chromogen for 10 min at ambient temperature, and (viii) hematoxylin counterstaining for 5 min at ambient temperature, followed by mounting of the slides. Normal human serum was used as a negative control. Histology slides were scanned with an Aperio ScanScope (Aperio Technologies, Vista, CA, USA). Immunostaining intensity was assessed in a blinded manner by two board-certified oncopathologists and scored on a scale of 0 to 3 (0 = negative, 1 = weak, 2 = moderate, and 3 = strong). The extent of staining was scored based on the percentage of stained cells (0 = negative, 1 = 1–25%, 2 = 26–50%, and 3 = 51–100%). Relative staining intensity was obtained by multiplying the intensity and extent scores (range, 0–9). Some samples with defective cores were not included in the analysis.

### Reverse transcription (RT)-PCR and quantitative real time (Q)-PCR

RT-PCR and Q-PCR were performed as described previously [44]. Total RNA was extracted using a TRIzol RNA extraction kit (Invitrogen). First-strand cDNA was synthesized with an iScript cDNA synthesis kit (Bio-Rad, Richmond, CA, USA). RT-PCR and quantitative real-time-PCR (Q-PCR) were performed as described previously. The sequences of primers for RT-PCR and Q-PCR were: forward CCR1, 5′-gccttctggttttatggg-3′; reverse CCR1, 5′-ctcctagacacttttcctc-3′; CCR1 TaqMan probe, 5′-FAM- agttccgactgccatcttggactttg-BHQ-3′; forward GAPDH, 5′-tcgacagtcagccgcatcttc-3′; reverse GAPDH, 5′-cgcccaatacgaccacct ccg-3′; and GAPDH TaqMan probe, 5′-Yakima Yellow TM-cgtcgccagcccagccacgc-BHQ-1-3′. Expression values were normalized to GAPDH mRNA using the software program provided by the manufacturer.

### Three-dimensional (3-D) spheroid invasion assay

A 3-D invasion assay was performed using the Cultrex 3-D Spheroid Cell Invasion Assay kit (Trevigen, Inc., Gaithersburg, MD, USA), as described previously [45]. MDA-MB-231 variant cells (3 × 10^3^ cells/well) expressing shRNA (shCT and shCCR1) were cultured for 3 days in Spheroid Formation Extracellular matrix to drive the aggregation and spheroid formation of cells followed by the addition of the Invasion Matrix composed of basement membrane proteins and medium for 1-5 days. The morphology of 3-D cell invasion was visualized using an Eclipse TS100 microscope equipped with a digital sight camera.

### Experimental pulmonary metastasis assay *in vivo*

Balb/c-nude female mice were obtained from Orient Bio Inc. (Seungnam, Republic of Korea). All animal experiments were performed at Gachon University following IACUC (Institutional Animal Care and Use Committee)-approved protocols (No.LCDI-2012-0069). For the experimental metastatic model, MDA-MB-231-luc cells (shCT and shCCR1, 1 x 10^6^ cells in 100 μl PBS) were injected into the lateral tail veins of 7-week-old Balb/c-nude mice (*n* = 10 each group). After 8 weeks, D-luciferin (15 mg/kg; PerkinElmer, Waltham, MA, USA) was intraperitoneally injected 10 min prior to imaging acquisition. To monitor pulmonary metastasis, *in vivo* bioluminescent imaging was conducted once a week using the Spectrum IVIS (PerkinElmer, Waltham, MA, USA). The BLI signal intensity (photons/sec) was quantified and calculated as the sum of all detected regions per second using Living Image software (Xenogen, Alameda, CA, USA).

### Immunoblot analysis

Cells were lysed in a buffer consisting of 20 mM HEPES (pH 7.2), 1% Triton X-100, 10% glycerol, 150 mM NaCl, 10 μg/mL leupeptin and 1 mM PMSF. The protein extracts (20 μg each) were separated by SDS-polyacrylamide gel electrophoresis and transferred to nitrocellulose membranes. Primary antibody against glyceraldehyde 3-phosphate dehydrogenase (GAPDH; 1:500) was obtained from Santa Cruz Biotechnology (Santa Cruz, CA, USA). Primary antibodies against phospho-Erk1/2 (Thr202/Tyr204; 1:1000), Akt1 (1:1000), phospho-Akt (Ser473; 1:1000), phospho-Stat3 (Tyr705; 1:500), and phospho-Stat3 (Ser727; 1:500), and phospho-mTOR (Ser2481; 1:1000) were purchased from Cell Signalling Technology (Danvers, MA, USA). The blots were incubated with the appropriate primary and secondary antibodies and developed using an enhanced chemiluminescence detection system (GE Healthcare, Piscataway, NJ, USA).

### CCR1 surface expression analysis via flow cytometry

CCR1 expression on the cell surface was determined using the FlowCellect Chemokine Receptor CCR1 Surface Expression Identification kit (Merck Millipore, Darmstadt, Germany) according to the manufacturer’s instructions.

### Construction and mutagenesis of human CCR1 promoter-reporter constructs

A fragment of the human *CCR1* gene was amplified from human genomic DNA (Promega) via PCR. The Primer sequences used to amplify the human *CCR1* gene spanning nucleotides –986 to +27 (transcription start site numbered +1) were 5′-agcaatgccgtgaggaaggt-3′ (forward; –986F) and 5′-tgtttctggggctttctggg-3′ (reverse; +27R). The amplified PCR products were ligated into a T&A vector (RBC Bioscience), followed by digestion with *Kpn*I and *Bgl*II and subcloning into the luciferase reporter plasmid pGL4-basic (Promega), yielding pCCR1-Luc(–982/+27). A series of deletion constructs was generated using pCCR1-Luc(–986/+27) as a template. The forward primer sequences used to generate these constructs were 5′-agctgggatttgaacttgctc-3′ (−750 to +27), 5′-agttagttttgaggaacaggactca-3′ (–500 to +27), 5′-cactgccacccacctcaccca-3′ (–300 to +27), 5′- aaatcctcgcagttaacttgg-3′ (–200 to +27), and 5′-tttgtcccttcttgtcattt-3′ (–110 to +27). One reverse primer, +27R, was used for all deletion constructs. The amplified PCR products were ligated into the T&A vector and then ligated into the *Kpn*I and *Bgl*II sites of the pGL4-basic vector, yielding pCCR1-Luc(−750/+27), pCCR1-Luc(−500/+27), pCCR1-Luc(−300/+27), pCCR1-Luc(−200/+27), and pCCR1-Luc(−110/+27), respectively. Site-specific mutation of the STAT3 binding site within the CCR1 promoter (ttCCagga → ttTTagga) was performed with a QuickChange site-directed mutagenesis system (Stratagene) using pCCR1-Luc(−200/+27) as a template. All mutations were verified by DNA sequencing.

### Luciferase promoter reporter assay

The luciferase promoter reporter assay was performed as described previously [46]. Firefly luciferase activity was normalized to *Renilla* activity, and the relative amount of luciferase activity in the untreated cells was set to “1”.

### Electrophoretic mobility shift assay (EMSA)

EMSA was performed using a DNA-binding protein detection system kit (Affymetrix, Santa Clara, USA) with minor modifications, as described previously [46]. The sequence of the synthetic deoxyoligonucleotide probe corresponding to the STAT binding sequence was 5′-biotin-gttaacttggcttccaggaagtggc-3′.

### Lentiviral shRNA-mediated gene silencing

MDA-MB-231 cells were transduced with lentiviral particles targeting Akt1, Akt2, CCR1, mTOR, or STAT3 (MISSION^®^ shRNA; Sigma-Aldrich, St. Louis, MO, USA), according to the manufacturer’s instructions.

### Statistical analysis

The results were expressed as the mean ± standard deviation (SD). Statistical significance of tissue microarray data was analysed by paired two-tailed *t*-test, *in vivo* metastatic BL intensity data were by unpaired *t*-test, and other data were analyzed by one-way ANOVA followed by Sidak’s multiple comparisons test with the GraphPad Prism version 7.02 program (GraphPad Software, San Diego, CA, USA). A *p*-value < 0.05 was regarded as statistically significant.

## SUPPLEMENTARY MATERIALS FIGURES AND TABLE


