# Supplementary material for: C-C motif chemokine receptor 1 (CCR1) is a target of the EGF-AKT-mTOR-STAT3 signaling axis in breast cancer cells
Source: Oncotarget. 2017 Oct 10;8(55):94591–605. doi: 10.18632/oncotarget.21813 (PMC5706898; doi:10.18632/oncotarget.21813)
Supplement: Supplementary file 1 [file oncotarget-08-94591-s001.pdf]

C-C motif chemokine receptor 1 (CCR1) is a target of the EGF-AKT-mTOR-STAT3 signaling axis in breast cancer cells

SUPPLEMENTARY MATERIALS

Supplementary Table 1: Statistical analysis of CCR1 immunostaining intensity in breast tissue microarray data

	Normal	IDC	Normal vs IDC
Number of values	178	178	178
Minimum	0	0	-3
25% Percentile	0	3	1.5
Median	0	6	3
75% Percentile	3	8.25	6
Maximum	6	9	9
Mean RU	1.669	5.781	4.112
S.D.	1.886	2.513	2.879
S.E.M.	0.1413	0.1884	0.2158
Lower 95% CI	1.39	5.409	3.686
Upper 95% CI	1.947	6.153	4.538
Paired t test	P value		<0.0001
	P value summary		****
	Significantly different (P < 0.05)?		Yes
	One- or two-tailed P value?		Two-tailed
	t, df		t=18.81 df=177
Pairing effectivity	Correlation coefficient (r)		0.1594
	P value (one tailed)		0.0168
	P value summary		*
	Pairing significant?		Yes

IDC, breast invasive ductal carcinoma.
Normal, adjacent peripheral normal tissue.
RU, immunohistochemical staining intensity of CCR1 in relative unit.
S.D., standard deviation; S.E.M., standard error of mean.
CI, confidential interval.

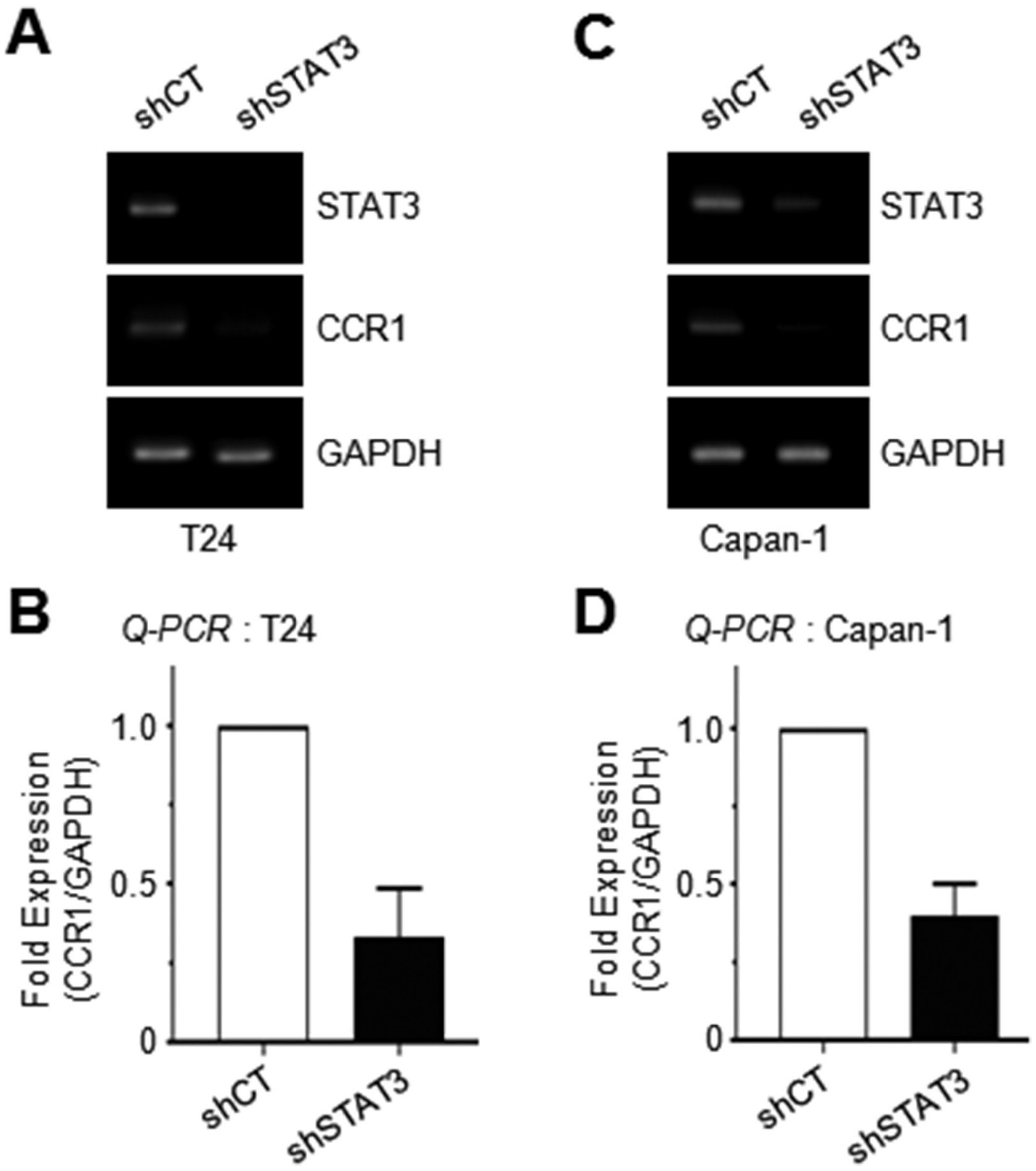

Supplementary Figure 1: Effect of STAT3 silencing on CCR1 expression. Total RNAs were extracted from T24 **(A** and **B)** and Capan-1 **(C** and **D)** cells and CCR1 mRNA levels were examined by RT-PCR (A and C) or quantitative real-time PCR (B and D). Data were presented as mean ± SD (*n* = 3).

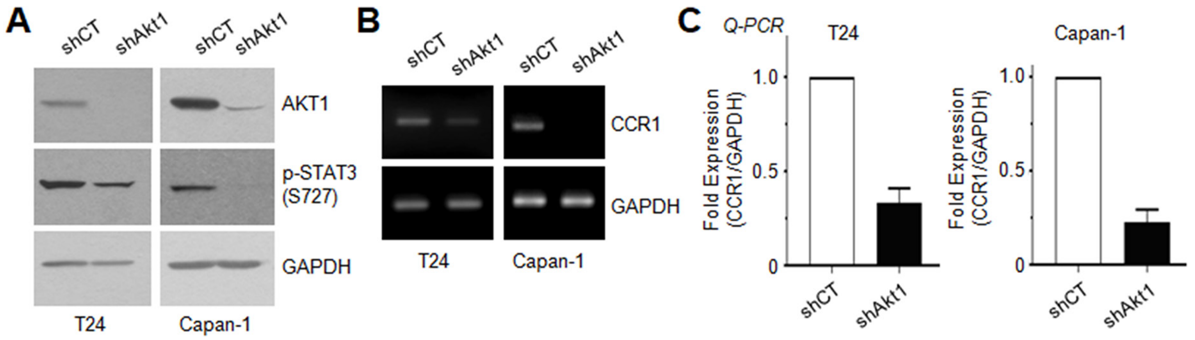

Supplementary Figure 2: Effect of AKT1 silencing on the serine-727 phosphorylation of STAT3. **(A)** Protein lysates were prepared from T24 or Capan-1 transfectants expressing control scrambled shRNA (shCT) or AKT1 shRNA (shAkt1). Immunoblot analysis was performed using AKT1 or phospho-STAT3 (Ser727) antibody. GAPDH was used as an internal control. **(B)** Total RNAs were extracted from T24 and Capan-1 transfectants, and CCR1 mRNA levels were examined by RT-PCR (B) or quantitative real-time PCR **(C)**. Data are presented as means ± SD (*n* = 3).

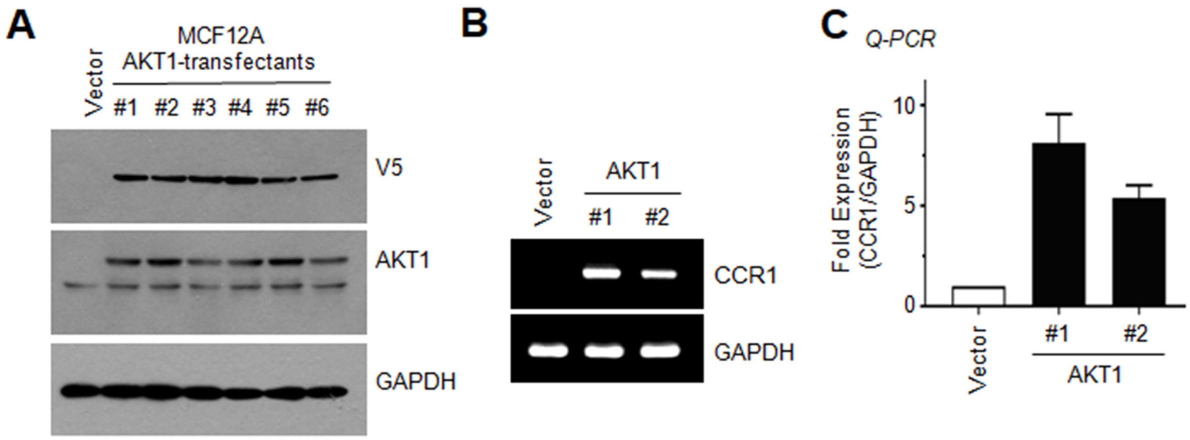

Supplementary Figure 3: Effect of AKT1 expression on CCR1 expression. **(A)** MCF12A cells were transfected with either empty vector or expression plasmid for V5-tagged AKT1 (clones #1 and -#2). Cell lysates were prepared, and immunoblotting was performed with antibodies against V5 and AKT1. GAPDH served as an internal control. **(B** and **C)** Total RNAs were extracted and CCR1 mRNA levels were examined in MCF12A transfectants expressing AKT1 (clones #1 and #2) by RT-PCR (B) or by quantitative real-time (Q)-PCR (C). Values were normalized to GAPDH mRNA levels. Data are presented as means ± SD (n = 3).
